# Right atrium area is associated with survival after out-of-hospital cardiac arrest: a single-center cohort study

**DOI:** 10.1186/s44156-025-00072-5

**Published:** 2025-04-14

**Authors:** King Hei Dominic Cheng, Samir Sulemane, Sara Fontanella, Petros Nihoyannopoulos

**Affiliations:** National Heart and Lung Institute, Hammersmith Hospital, Du Cane Road, London, W12 0NN UK

**Keywords:** Out-of-hospital cardiac arrest, Echocardiography, Right atrium area, Right atrial area, Global longitudinal strain, Survival, Cardioverter-defibrillator

## Abstract

**Background:**

Out-of-hospital cardiac arrest (OHCA) is associated with high mortality, highlighting the importance of identifying prognostic factors to guide treatment escalation plans. This study investigates the short-term prognostic potential of transthoracic echocardiogram (TTE), a commonly performed investigation in OHCA patients. This study is among the first to report left ventricle (LV) global longitudinal strain (LVGLS) in OHCA patients.

**Methods:**

This single-center retrospective cohort study included 54 patients treated between 2019 and 2022, during the COVID-19 pandemic. Patient characteristics were reported using the 2015 Utstein template, and echocardiographic parameters were assessed following British Society of Echocardiography guidelines. Univariate analyses compared TTE parameters by survival-to-discharge and implantable cardioverter-defibrillator implantation outcomes. Correlations between LV ejection fraction (LVEF) derived from cardiac magnetic resonance imaging (cMRI) and echocardiographic LV systolic parameters were evaluated.

**Results:**

The survival-to-discharge rate was 77.8%. Non-survivors had a significantly larger right atrium (RA) area (RAA) (20.8 cm^2^ vs. 15.2 cm^2^ in survivors; *p* = 0.003). No statistically significant differences were observed for other right or left heart parameters. The median LVGLS was reduced at -11.4% (interquartile range: -14.0 to -7.6). LVEF correlates well on cMRI and TTE (Pearson correlation coefficient = 0.830).

**Conclusion:**

This study identifies a novel association between larger RAA and short-term mortality following OHCA, alongside a higher survival rate in a tertiary center. Further research should consider incorporating RA parameters into analyses to refine prognostic assessments.

**Supplementary Information:**

The online version contains supplementary material available at 10.1186/s44156-025-00072-5.

## Introduction

Out-of-hospital cardiac arrest (OHCA) has a global average survival rate of 7% [1] and is associated with substantial in-patient costs, which increase with prolonged survival, including cases of eventual in-patient mortality [2]. Identifying patients likely to survive OHCA is essential for guiding treatment escalation and ensuring efficient use of healthcare resources.

Beyond survival, predicting the need for an implantable converter-defibrillator (ICD) is another clinically important consideration in OHCA management. ICD use is associated with improved five-year survival rates even in patients with more comorbidities [3], which themselves are linked to with poorer outcomes [4]. While the National Institute for Health and Care Excellence (NICE) [5] considers ICD use cost-effective, the upfront cost of approximately £15,000 underscores the need for predictive factors to optimise healthcare resource allocation for OHCA patients.

Cardiac imaging, particularly transthoracic echocardiography (TTE) and cardiac magnetic resonance imaging (cMRI), plays an indispensable role in the evaluation of OHCA patients. This is because cardiac pathologies are not only major causes of OHCA [4, 6], but also key components of post-resuscitation myocardial dysfunction (PRMD), which complicates recovery [7]. While the current role of cardiac imaging resides in diagnosing the causes and complications of OHCA, its utility would be enhanced if imaging findings also offer prognostic insights.

Few studies have explored the direct relationship between cardiac imaging parameters and survival following a cardiac arrest. Among these, echocardiographic left ventricle (LV) ejection fraction (LVEF), a widely studied parameter for the prognostication of other cardiac conditions, has shown conflicting results [8–14]. A recent scoping review of 11 studies found no association between LVEF and survival or neurologic outcomes [15], likely due to the confounding effect of PRMD, which transiently reduces LVEF [8–9]. To date, no study has assessed the association between short-term outcomes after cardiac arrest and LV global longitudinal strain (LVGLS), an alternative measure of LV systolic function with advantages such as reduced operator dependency and lower intraobserver variability [16]. Given its established prognostic value in predicting all-cause mortality across various conditions [17], investigating LVGLS in the context of OHCA is warranted.

Focusing on the right ventricle (RV), Ramjee et al. [18] reported that RV dysfunction was associated with increased mortality after OHCA. Based on the RV myocytes’ distinct capacity to increase oxygen extraction during ischaemia and its continuous perfusion throughout the cardiac cycle supported via an extensive collateral system, they reasoned that RV dysfunction after OHCA may indicate severe ischaemic injury. Patel et al. [19] corroborated these findings, linking RV dysfunction to haemodynamic instability in cardiac arrest patients undergoing percutaneous coronary intervention (PCI) and reporting a consistent association between RV dysfunction and mortality.

Notably, none of these studies were conducted in the United Kingdom (UK), which features a unique healthcare system, the National Health Service (NHS), and its own national echocardiography guidelines published by the British Society of Echocardiography (BSE) [20–22]. Thus, global findings may not fully apply to the UK context.

This study primarily aims to investigate the association between TTE parameters and short-term survival and/or ICD implantation rates in OHCA patients. Based on the existing literature, we hypothesise that RV dysfunction is associated mortality, whereas LV systolic dysfunction is not.

## Methods

This is a single-center retrospective cohort study spanning a 32-month period during the COVID-19 pandemic, from September 2019 to April 2022 at the Hammersmith Hospital (HH), an academic tertiary center with primary PCI services.

Under standard care, OHCA patients within the catchment area are transported by the London Ambulance Service (LAS), the local emergency medical service (EMS), to HH. These patients typically arrive at the Heart Assessment Clinic (HAC) for initial resuscitation, where the screening of this study took place. Patients with a pre-hospital diagnosis of cardiac arrest were screened in. However, only those who underwent a formal TTE during their index admission, defined as being reviewed and reported by a BSE-accredited personnel, were included to ensure that the TTE performed met national standards. TTE images were reviewed, and patients were excluded if a standard parasternal long axis (PLAX) view and/or an apical 4-chamber (A4C) view were not obtained, as these windows are essential components of a comprehensive TTE assessment. Patients with return of spontaneous circulation (ROSC) prior to the arrival of the LAS were excluded, as bystanders may have misdiagnosed cardiac arrest in these cases. Finally, patients with a non-medical pathogenesis of OHCA, as defined by the 2015 Utstein template [23], the international consensus on reporting format for OHCA registries and studies [24], were excluded to standardise the pathophysiology of OHCA and reduce data variability in this small sample study. Since medical aetiologies account for the majority of OHCA [4, 6], resulting biases would be minimal.

Non-radiographical patient data was collected in accordance with the 2015 Utstein template. The core domains of the template were collected and presented as far as feasible to comply with international reporting standards. The primary and secondary outcome were survival to discharge and ICD implantation within the index admission, respectively.

TTE parameters on the first formal TTE performed during the index admission were determined and interpreted based on the latest BSE guidelines where applicable [20–22]. When available, parameters recorded in the report were used. Parameters not routinely reported under the local protocol were measured by a single investigator blinded to the outcome, except for visually estimated LVEF and wall motion score (WMS), which were determined by a single cardiology expert blinded to patient outcomes due to the expertise required. For parameters not included in the guidelines, well-established formulas were used. The cube formula determines LV mass, and a 16-segment model determines WMS and WMS index (WMSI) [25]. Stroke volume was determined as the product of 0.785, the velocity time integral (VTI) of the LV outflow tract (LVOT), and the square of the LVOT diameter [26].

All LVGLS analyses were performed with TomTec Arena TTA2.41 Build 514,944 (Germany) [27], by a single investigator blinded to patient outcomes.

cMRI LVEF was extracted from the radiology report. cMRI was only performed in patients for whom it was clinically feasible and indicated.

### Statistical analysis

The Shapiro-Wilk test (SW test) was used to test for normality of the investigated variables in each outcome subgroups.

Categorical data were evaluated between outcome subgroups using the Fisher’s exact test to correct for small expected counts. Continuous data were analysed with the non-parametric Mann-Whitney U test when the values were non-normally distributed, as well as WMS and WMSI. The exact p-value, compared to a significance level of 0.05, was adopted. Normally distributed data were analysed using the t-test for independent samples.

Correlation analyses were performed between TTE and cMRI LVEF using the Pearson correlation coefficient (PCC). Of note, all patients who had a cMRI died.

All statistical tests were performed with STATA/BE 17.0 for Windows Revision 10 May 2022 (United States of America) [28], unless otherwise stated. The PCC was derived using the CI2 STATA module (United Kingdom) [29] when normality could not be ruled out by a SW test.

## Results

**Fig. 1:**
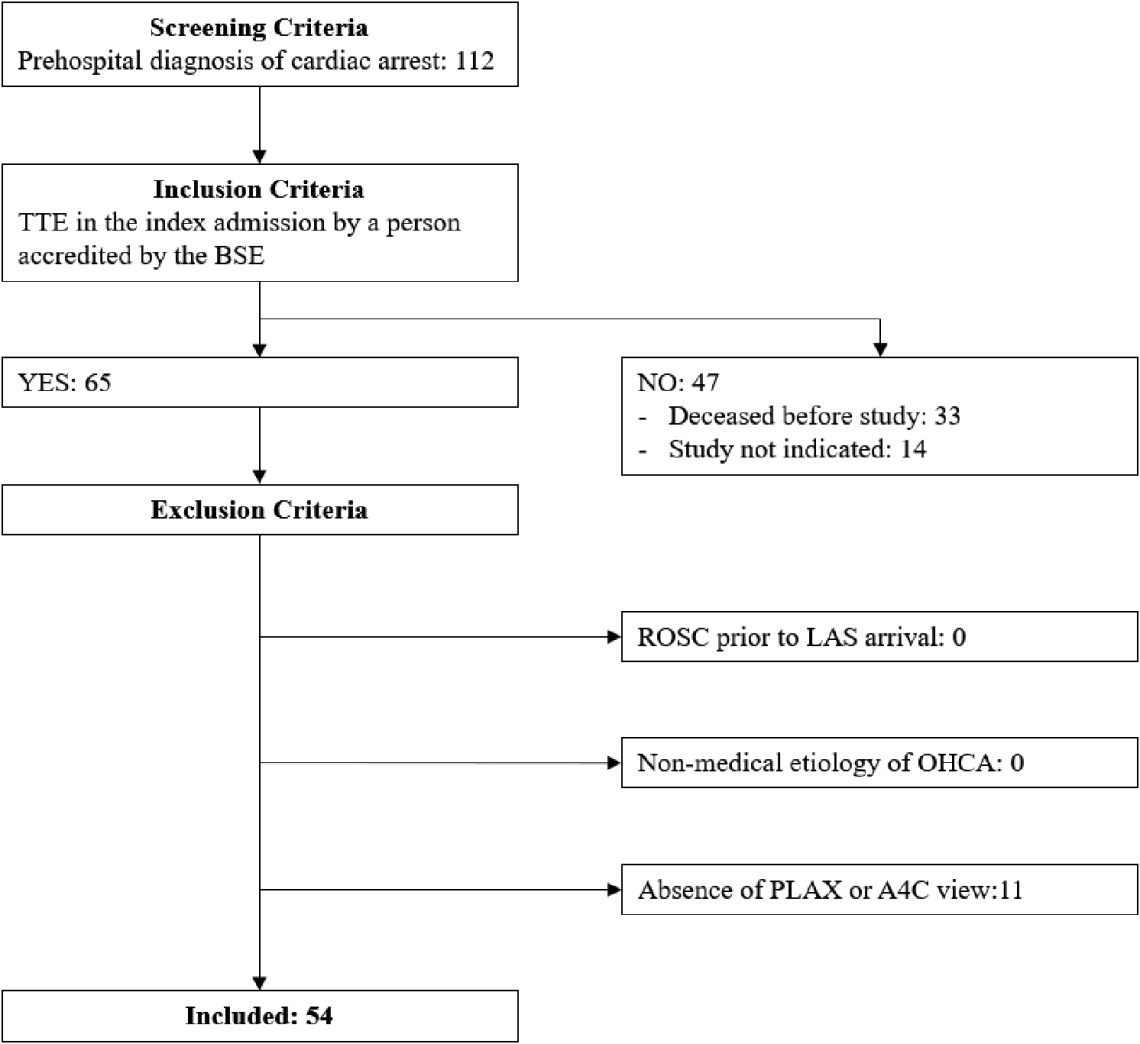
Patient selection flow diagram

Figure 1 summarises the patient selection process. Of the 112 patients admitted to the HAC during the study period, fewer than half (54; 48%) were included. Thirty-three (29%) died before an echocardiogram was done, while an echocardiogram was not indicated for the other 14 (13%) patients for reasons including a decision for a palliative care approach or a cMRI being performed instead. Eleven were further excluded due to a lack of PLAX and/or A4C view(s).

Tables 1 and 2 and additional files 1 and 2 show the characteristics of the patients in the study population overall, by survival-to-discharge, and by ICD implantation. The mean values are accompanied by one standard deviation (SD) in brackets, while the median values are accompanied by the interquartile range (IQR).

**Table 1:** Continuous variables of patient characteristics by survival-to-discharge

	Total (*N* = 54)				Survivor (*N* = 42)				Non-survivor (*N* = 12)				
Patient characteristic (Continuous variables)	Number of known data		Value from known data		Number of known data		Value from known data		Number of known data		Value from known data		Comparison *p*-value
		%	Mean Median	1SD IQR		%	Mean Median	1SD IQR		%	Mean Median	1SD IQR	
Age (years)	54	100	60.3	± 11.2	42	100	58.4	± 10.5	12	100	66.8	± 11.6	0.021*
Downtime (mins)	45	83.3	18.6	± 12.0	36	85.7	17.3	± 12.2	9	75	23.8	± 10.5	0.067^a^
			17	8–24			15	8-23.5			24	19–30	
Activation to EMS arrival (mins)	14	25.9	6.35	± 3.25	7	16.7	6.57	± 3.69	7	58.3	6.14	± 3.02	0.816
Activation to defibrillation (mins)	11	20.4	5.55	± 3.33	7	16.7	5.14	± 3.63	4	33.3	6.25	± 3.10	0.622
Arrest to cardiopulmonary	37	68.5	1.46	± 3.30	27	64.3	1.3	± 3.34	10	83.3	1.9	± 3.34	0.613^a^
Resuscitation (CPR) (mins)			0	0–0			0	0–0			0	0–3	
Initial pH	41	75.9	7.26	± 0.127	29	69.0	7.27	± 0.12	12	100	4.78	± 2.51	0.356
Initial lactate (mmol/L)	41	75.9	4.0	± 2.77	29	69.0	3.68	± 2.84	12	100	3.33	± 5.07	0.136^a^
			3	1.7–6.2			2.4	1.6–5.1			4.65	2.9–6.6	

This table shows the continuous variables of patient characteristics for the overall study population, survivors and non-survivors. The first column of each patient group presents the number of known data values for each variable, along with the percentage out of the total number of patients in a group. The mean and SD for each variable in each group are provided. When the SW test p-value is < 0.05 in any subgroup, the median and the IQR are also given. The comparison p-value is obtained using the t-test for independent samples, or for values marked with (^a^), using the exact probability from a Mann-Whitney U test. An asterisk (*) indicates a statistical significance when *p* < 0.05.

**Table 2:** Continuous variables of patient characteristics by ICD implantation in index admission

	Total (*N* = 54)					ICD-implantation (*N* = 16)					Non-ICD-implantation (*N* = 38)					
Characteristic	Number of known data		Value from known data		Number of known data			Value from known data		Number of known data			Value from known data		Comparison *p*-value	
		%	Mean Median	1SD IQR			%	Mean Median	1SD IQR			%	Mean Median	1SD IQR		
Age (years)	54	100	60.3	± 11.2	16		100	56.9	± 10.6	38		100	61.7	± 11.3	0.149	
Downtime (mins)	45	83.3	18.6	± 12.0	14		87.5	15.8	± 8.84	31		81.6	19.9	± 13.1	0.470^a^	
			17	8–24				14.5	8–23				18	8–30		
Activation to EMS arrival (mins)	14	25.9	6.35	± 3.25	3		18.8	5.67	± 4.04	11		28.9	6.55	± 3.21	0.626^a^	
			7	3–9				8	1–8				6	3–10		
Activation to defibrillation (mins)	11	20.4	5.55	± 3.33	3		18.8	5.67	± 4.04	8		21.1	5.5	± 3.34	0.994^a^	
			6	2–8				8	1–8				5.5	2.5–8.5		
Arrest to CPR (minutes)	37	68.5	1.46	± 3.30	10		62.5	1.7	± 3.34	27		71.1	1.37	± 3.35	0.700^a^	
			0	0–0				0	0–1				0	0–0		
Initial pH	41	75.9	7.26	± 0.127	12		75.0	7.21	± 0.0791	29		76.3	7.28	± 0.138	0.102	
Initial lactate (mmol/L)	41	75.9	4.0	± 2.77	12		75.0	4.87	± 2.73	29		76.3	3.64	± 2.75	0.169^a^	
			3.0	1.1–6.2				5.05	2.25–6.5				2.8	1.6–4.9		

This table shows the continuous variables of patient characteristics for the overall study population, and patients with and without ICD implantation. The first column of each patient group presents the number of known data values for each variable, along with the percentage out of the total number of patients in the group. The mean and the SD for each variable in each group are provided. When the SW test p-value is < 0.05 in any subgroup, the median and the IQR are also given. The comparison p-value is obtained using the t-test for independent samples, or for values marked with (^a^), using the exact probability from a Mann-Whitney U test.

The study population had a mean age of 60.3 (± 11.2) years and was primarily composed of male patients (45; 83.3%). Age was significantly higher (*p* = 0.021) among the non-survivors (66.8 ± 11.6) compared to the survivors (58.4 ± 10.5). Non-survivors had significantly higher frequencies of atrial fibrillation (Afib) (*p* = 0.046) and chronic obstructive pulmonary disease (COPD) (*p* = 0.031). None of the patients had a history of ICD implantation prior to the index admission.

All OHCA were due to medical causes, with STEMI accounting for over half (29; 53.7%) of the cases. Most of the events were witnessed (46; 90.2%) and presented as an initial shockable cardiac rhythm (46; 86.8%), all of which were managed with defibrillation. Around three-quarters (33; 74.4%) received bystander CPR, and about one-quarter (12; 28.6%) received bystander defibrillation.

The majority (52; 96.3%) received a coronary angiogram, with PCI performed in 29 (53.7%) of them. Forty-two (77.8%) survived to discharge, and 16 (29.6%) had an ICD implantation. None of the non-survivors received an ICD, whereas 38.1% (16/42) of survivors did (*p* = 0.011).

Between the two ICD implantation groups, fewer statistically significant differences were observed. None of the patients who received ICD implantation had diabetes mellitus (DM), whereas 34.2% (13) of those in the non-ICD implantation group had (*p* = 0.006). The most frequent locations of arrest were sports or recreation events (35.7%; 5/14) in the ICD-implantation group and public buildings (43.3%; 13/30) in the non-ICD-implantation group (*p* = 0.030).

Additional files 3–4 show the echocardiographic parameters of the patients in the study population overall, by survival-to-discharge, and by ICD implantation. The mean values are accompanied by one SD in brackets, while the median values are accompanied by the IQR.

Notably, the non-survivors received an echocardiogram significantly earlier than the survivors, with the non-survivors exhibiting less variability (median of 2 [IQR: 1–3] days after OHCA compared to 5.5 [IQR: 2–10] in survivors; *p* = 0.021). All but one (91.7%) non-survivor received an echocardiogram within five days of admission, compared with only 42.9% (18/42) of the survivors.

Of the echocardiographic parameters, right atrium area (RAA) was the only one that differed significantly between the two survival groups (*p* = 0.003). Non-survivors had a higher mean RAA (20.8 ± 4.84cm^2^) than survivors (15.2 ± 4.46cm^2^).

Twenty-three (69.7%) patients had normal RA pressure. The mean RV systolic pressure, tricuspid annular plane systolic excursion (TAPSE), and fractional area change (FAC) were normal at 36.1 ± 13.2mmHg, 1.95 ± 0.0500 cm, and 41.7 ± 10.2% respectively. Mean LVFS was 27.7 ± 9.97%; median WMSI was 1.6 (IQR: 1.3–1.9), suggestive of mild hypokinesia [30]; mean LVEF was impaired at 43.9 ± 11.4%; and median peak averaged LVGLS was reduced at -11.4% (IQR: -7.61 to -14.0).

No statistical significance regarding the echocardiographic parameters was observed in the study population by ICD implantation.

Thirteen patients had a cMRI scan. Correlation between TTE and cMRI LVEF was good based on the four-grade model [31], with a PCC of 0.830.

## Discussion

The patient characteristics in this study are best compared with data published by the UK’s national OHCA registry, the OHCA Outcome Registry, particularly with the data from the LAS 2020 report [32]. Notably, the gender and age distributions in this study were similar to those in the report, suggesting that our sample represents the typical OHCA population in London.

The most striking finding was an exceptionally high survival rate of 77.8%, compared to just 26.5% in the report. While it might be a result of including only patients who survived until a TTE assessment, the rate remained higher at 37.5% when the entire screened population was included (42/112). A possible explanation is the specialised nature of the study center as a primary PCI center. This theory is supported by the near 100% coronary angiogram rate and the high incidence of STEMIs, which might have prompted preferential referrals of these patients to the center. Prompt and successful PCIs, performed in half of the patients, have been shown to improve survival [33]. Several other characteristics that favoured survival were also found in a higher proportion of the study population compared to the LAS report, including a shockable first cardiac rhythm [1, 4, 7, 34], witnessed arrest, and the presence of prompt bystander CPR and defibrillation [1, 34–35].

The differences between ICD implantation groups reflected the clinical considerations for ICD implantation. There were concerns about higher risks of complications and mortality with ICD use in diabetic patients [36–37], which may have been shared by the clinicians in the study center. OHCA occurring at sports events might have raised suspicion for an exercise-induced aetiology, for which ICD implantation is a logical management strategy. Notably, no patients had hypertrophic cardiomyopathy based on the anatomic distribution of hypertrophy and family history.

Multiple echocardiographic parameters suggested an impaired LV systolic function in the study population. These findings corelated with cMRI LVEF and were consistent with the literature, potentially representing the entity of PRMD [8–10]. This theory is further supported by the reduced hemodynamic parameters observed. In contrast, LV diastolic parameters were generally within the normal range, differing from studies that consistently observed these parameters as the strongest predictor of survival from OHCA [9–10]. The similarity in LV systolic function between survival groups is consistent with the literature [15].

Surprisingly, RAA was the only echocardiographic parameter that differed significantly between the survival groups, even though the mean values remained within the normal range. The finding was unusual, as it occurred in isolation from other right heart parameters. To our knowledge, this is a novel finding that has not been reported.

Although increased RAA could be attributed to chronic atrial fibrillation, which was not present in the survivor group, it was only present in 16.7% (2/12) of non-survivors. Similarly, while one might suspect the increased RAA to be attributed to a higher incidence of COPD in non-survivors, it is noteworthy that 75% (3/4) of COPD patients in this study had missing RAA values, and the remaining one survived. Hence, another explanation is warranted.

An animal study showed that RA volume and contractility increase along with RV diastolic dysfunction in chronic RV pressure overload, hypothesised as a compensatory mechanism to maintain cardiac output [38]. The larger RAA in our study may reflect compensation for RV diastolic dysfunction, possibly increasing vulnerability to decompensation and cardiac death. In fact, an enlarged RAA has been found to be associated with poor outcomes including death in a meta-analysis on pulmonary arterial hypertension [39] and studies on heart failure [40–41]. Our finding on RAA aligns with the patterns observed in the literature and demonstrates a potential prognostic marker within the standard TTE procedures, as RAA could be easily measured on a standard RV A4C view. We suggest future prognostic TTE studies on OHCA to measure and report RAA.

Paradoxically, it was reassuring to observe a similarity in echocardiographic parameters between the ICD implantation groups. This suggests that cardiac dysfunction per se did not favor or deter ICD implantation, a procedure shown to improve overall survival even among patients with a lower LVEF on discharge [3]. This finding might support the approach of centering the decision of ICD implantation on clinical grounds, rather than on seemingly alarming echocardiographic findings.

## Limitations

The maximal power for the statistical tests across all echocardiographic variables in the current study (G*Power Version 3.1.9,6 [42]) was 0.31 for survival groups and 0.36 for ICD implantation groups. The low power and the lack of expected statistically significant trends in patient characteristics known to predict survival suggest the presence of a type 2 error. This issue is exacerbated by the inclusion of only patients who had a complete and formal TTE, intended to ensure the accuracy and completeness of the echocardiographic parameter dataset. Patients with early mortality or some critically ill patients who could not be manipulated for image optimisation were excluded. Consequently, this study may have failed to identify parameters associated with rapid deterioration and mortality. Nevertheless, the results indicate that RAA may be an important predictor of survival in OHCA patients.

However, right heart parameters, including RAA, might be affected by mechanical ventilation secondary to the increase in RV afterload [43–44], and by the fluctuations in haemodynamics in critically ill OHCA patients. The use of mechanical ventilation is likely more frequent in non-survivors, probably for airway protection in the presence of severe neurological damage, a major cause of mortality following OHCA [7]. While we found no statistically significant differences between the two groups in mean arterial pressure, stroke volume index, and cardiac index, other measurements of hemodynamics, such as central venous pressure, may help to clarify its impact on RAA. Further research that reports on mechanical ventilation use, neurological outcomes, and additional haemodynamic measures is warranted.

The difference in the timing of echocardiography might have confounded the study. Echocardiography was prioritised for critically ill patients. Studies have shown that some echocardiographic parameters, such as LVEF, peak early inflow velocity (E), peak early annular velocity (e’) and cardiac output (CO), change over time following OHCA [8–9]. An earlier echocardiogram in the non-survivors might have detected early PRMD, which could also have been present but resolved in the survivor group. Therefore, differences detected in the non-survivors might be a manifestation of PRMD rather than representing an association with mortality.

The technically challenging TTE in critically ill patients also constrained sector depth optimisation and compromised the frame rate. On some echocardiograms, it was below 40 frame-per-second, the minimum frame rate required for LVGLS analysis [21]. Along with the prominent noise on the images, the LVGLS analyses may have been inaccurate.

Echocardiograms in this study were performed by different BSE-accredited sonographers using various ultrasound systems for practical reasons. For instance, a portable point-of-care system might have been used when patient transport was deemed inappropriate. Hence, interobserver and intervendor variability in the acquisition and measurement of echocardiographic parameters was unavoidable.

Caution must be taken that this study was conducted during the COVID-19 pandemic, during which OHCA outcomes have been shown to be worse as compared to previous baseline values [45–46], and mechanical ventilation use may be more frequent. This impact on the study’s validity was balanced with the necessity of aligning the study’s OHCA systems and management approaches with current ones, such as the establishment of primary PCI services in the UK [47].

This study was conducted at a specialised primary PCI center, which was designated as a “cardiac arrest center (CAC)” in the ARREST trial [48], the study period of which overlapped with the current study. While non-STEMI patients were randomised to CAC or non-CAC in the trial, patients with suspected STEMI were referred to a primary PCI center. Patients with non-cardiac aetiology might also have been diverted to other centers, as suggested by the absence of these patients in the population meeting the inclusion criteria. This results in another selection bias. Conducting a similar study in a non-CAC may shed light on the differences between the two centers and the generalisability of this study.

Given the limitations, the novel finding of a higher RAA in non-survivors requires confirmation through future studies. A prospective study with TTE performed promptly upon patient arrival at the hospital could overcome the selection biases, the confounding effect of the variable timing of TTE, and interobserver and intervendor variability. This approach would allow the acquisition of dedicated images for speckle tracking analysis to investigate RA strain as a part of a comprehensive evaluation on RA parameters, and to reexamine the prognostic potential of LV systolic dysfunction considering the inconsistent findings in the literature regarding LVEF. The collection of data on addition haemodynamic measures would also be feasible.

## Conclusion

This study highlights a novel association between increased RAA and short-term mortality following OHCA, alongside a higher survival rate in a tertiary center. However, neither echocardiographic LVEF nor LVGLS was associated with survival. This study also underscores the success of a primary PCI center managing OHCA. Finally, its methodology provides a foundation for future echocardiographic research on OHCA patients.

## Electronic supplementary material

Below is the link to the electronic supplementary material.

Supplementary Material 1: **Additional file 1**: Categorical variables of patient characteristics by survival-to-discharge.

Supplementary Material 2: **Additional file 2**: Categorical variables of patient characteristics by ICD implantation in index admission.

Supplementary Material 3: **Additional file 3**: Echocardiographic characteristics by survival-to-discharge.

Supplementary Material 4: **Additional file 4**: Echocardiographic characteristics by ICD implantation in index admission.

## Data Availability

The datasets generated during the current study are not publicly available due to patient privacy.

## Abbreviations

95%CI: 95% confidence interval
A4C: Apical 4-chamber
ADL: Activities of daily living
AED: Automated external defibrillator
Afib: Atrial fibrillation
BSA: Body surface area
BSE: British Society of Echocardiography
CAC: Cardiac arrest center
CAD: Coronary arterial disease
cMRI: Cardiac magnetic resonance imaging
CO: Cardiac output
COPD: Chronic obstructive pulmonary disease
CI: Cardiac index
CPR: Cardiopulmonary resuscitation
DM: Diabetes mellitus
DT: Deceleration time
E: Peak early inflow velocity
e’: Peak early annular velocity
E/e’: Peak early inflow to annular velocity ratio
E/A: Peak early inflow to late inflow velocity ratio
EMS: Emergency medical service
FAC: Fractional area change
HAC: Heart Assessment Clinic
HH: Hammersmith Hospital
HR: Heart rate
HT: Hypertension
ICC: Intraclass correlation coefficient
ICD: Implantable cardioverter-defibrillator
IQR: Interquartile range
LA: Left atrium
LAS: London Ambulance Service
LAV: Left atrium volume
LAVI: Left atrium volume index
LV: Left ventricle
LVEDD: Left ventricle end-diastolic diameter
LVEF: Left ventricle ejection fraction
LVESD: Left ventricle end-systolic diameter
LVFS: Left ventricle fractional shortening
LVGLS: Left ventricle global longitudinal strain
LVOT: Left ventricle outflow tract
MAP: Mean arterial pressure
NHS: National Health Service
NICE: National Institute for Health and Care Excellence
OHCA: Out-of-hospital cardiac arrest
PCC: Pearson’s correlation coefficient
PCI: Percutaneous coronary intervention
PEA: Pulseless electrical activity
PLAX: Parasternal long axis
PRMD: Post-resuscitation myocardial dysfunction
pVT: Pulseless ventricular tachycardia
RA: Right atrium
RAA: Right atrium area
RAP: Right atrium pressure
ROSC: Return of spontaneous circulation
RV: Right ventricle
RVSP: Right ventricle systolic pressure
s’: Peak systolic annular velocity
SD: Standard deviation
SDp: Pooled standard deviation
STEMI: ST-segment elevation myocardial infarction
SV: Stroke volume
SVI: Stroke volume index
SW test: Shapiro-Wilk test
TAPSE: Tricuspid annular plane systolic excursion
TIA: Transient ischemic attack
TTE: Transthoracic echocardiogram
TTM: Therapeutic/targeted temperature management
UK: United Kingdom
Vfib: Ventricular fibrillation
Vmax: Peak velocity
VTI: Velocity time integral
WMS: Wall motion score
WMSI: Wall motion score index
