# Supplementary material for: Right atrium area is associated with survival after out-of-hospital cardiac arrest: a single-center cohort study
Source: Echo Res Pract. 2025 Apr 14;12:9. doi: 10.1186/s44156-025-00072-5 (PMC11995584; doi:10.1186/s44156-025-00072-5)
Supplement: Supplementary file 2 — Supplementary Material 2: Additional file 2: Categorical variables of patient characteristics by ICD implantation in index admission. [file 44156_2025_72_MOESM2_ESM.docx]

Additional file 2: Categorical variables of patient characteristics by ICD implantation in index admission

|  | **Total (N=54)** | | | | | | **ICD-implantation (N=16)** | | | | | | **Non-ICD-implantation (N=38)** | | | | | |  |
| --- | --- | --- | --- | --- | --- | --- | --- | --- | --- | --- | --- | --- | --- | --- | --- | --- | --- | --- | --- |
| **Characteristic** | **Number of known data** | | | **Value from known data** | | | **Number of known data** | | | **Value from known data** | | | **Number of known data** | | | **Value from known data** | | | **Comparison**  **p-value** |
|  |  | **%** | |  | **%** | |  | **%** | |  | **%** | |  | **%** | |  | **%** | |  |
| ICD implantation | 54 | 100 | | 16 | 29.6 | |  |  | |  |  | |  |  | |  |  | |  |
| Survival-to-discharge | 54 | 100 | | 42 | 77.8 | | 16 | 100 | | 16 | 100 | | 38 | 100 | | 26 | 68.4 | | 0.011* |
| Baseline Condition |  |  | |  |  | |  |  | |  |  | |  |  | |  |  | |  |
| Male | 53 | 98.1 | | 45 | 83.3 | | 16 | 100 | | 14 | 87.5 | | 37 | 97.4 | | 31 | 83.8 | | 1.000 |
| ADL independent | 52 | 96.3 | | 51 | 98.1 | | 16 | 100 | | 15 | 93.8 | | 36 | 94.7 | | 36 | 100 | | 0.308 |
| ICD implantation | 54 | 100 | | 0 | 0 | | 0 | 0 | | 0 | 0 | | 0 | 0 | | 0 | 0 | |  |
| Comorbidity present | 54 | 100 | | 38 | 70.4 | | 16 | 100 | | 9 | 56.3 | | 38 | 100 | | 29 | 76.3 | | 0.194 |
| Afib |  |  | | 2 | 3.7 | |  |  | | 0 | 0 | |  |  | | 2 | 5.3 | | 1.000 |
| Asthma |  |  | | 5 | 9.3 | |  |  | | 0 | 0 | |  |  | | 5 | 13.2 | | 0.306 |
| CAD |  |  | | 15 | 27.8 | |  |  | | 5 | 31.3 | |  |  | | 10 | 26.3 | | 0.747 |
| COPD |  |  | | 4 | 7.4 | |  |  | | 0 | 0 | |  |  | | 4 | 10.5 | | 0.306 |
| Chronic kidney disease |  |  | | 2 | 3.7 | |  |  | | 0 | 0 | |  |  | | 2 | 5.3 | | 1.000 |
| Depression |  |  | | 3 | 5.6 | |  |  | | 0 | 0 | |  |  | | 3 | 7.9 | | 0.547 |
| DM |  |  | | 13 | 24.1 | |  |  | | 0 | 0 | |  |  | | 13 | 34.2 | | 0.006* |
| Heart failure |  |  | | 1 | 1.9 | |  |  | | 0 | 0 | |  |  | | 1 | 2.6 | | 1.000 |
| HT |  |  | | 14 | 25.9 | |  |  | | 2 | 12.5 | |  |  | | 12 | 31.6 | | 0.187 |
| Obstructive sleep apnea |  |  | | 2 | 3.7 | |  |  | | 0 | 0 | |  |  | | 2 | 5.3 | | 1.000 |
| Peripheral arterial disease |  |  | | 2 | 3.7 | |  |  | | 0 | 0 | |  |  | | 2 | 5.3 | | 1.000 |
| Pulmonary embolism |  |  | | 1 | 1.9 | |  |  | | 0 | 0 | |  |  | | 1 | 2.6 | | 1.000 |
| Stroke/TIA |  |  | | 1 | 1.9 | |  |  | | 0 | 0 | |  |  | | 1 | 2.6 | | 1.000 |
| Valvular heart disease |  |  | | 1 | 1.9 | |  |  | | 1 | 6.3 | |  |  | | 0 | 0 | | 0.296 |
| Arrest |  |  | |  |  | |  |  | |  |  | |  |  | |  |  | |  |
| Medical cause | 53 | 98.1 | | 53 | 100 | | 15 | 93.8 | | 15 | 100 | | 38 | 100 | | 38 | 100 | |  |
| STEMI | 53 | 98.1 | | 29 | 53.7 | | 16 | 100 | | 6 | 37.5 | | 38 | 100 | | 23 | 60.5 | | 0.145 |
| Arrest location | 44 | 81.5 | |  |  | | 14 | 87.5 | |  |  | | 30 | 78.9 | |  |  | | 0.030* |
| Home |  |  | | 12 | 27.3 | |  |  | | 4 | 28.6 | |  |  | | 8 | 26.7 | |  |
| Workplace |  |  | | 7 | 15.9 | |  |  | | 3 | 21.4 | |  |  | | 4 | 13.3 | |  |
| Sports/ recreation event |  |  | | 6 | 13.6 | |  |  | | 5 | 35.7 | |  |  | | 1 | 3.3 | |  |
| Streets/ Highway |  |  | | 3 | 6.8 | |  |  | | 0 | 0 | |  |  | | 3 | 10 | |  |
| Public building |  |  | | 15 | 34.1 | |  |  | | 2 | 14.3 | |  |  | | 13 | 43.3 | |  |
| Assisted living/nursing home |  |  | | 0 | 0 | |  |  | | 0 | 0 | |  |  | | 0 | 0 | |  |
| Educational institution |  |  | | 0 | 0 | |  |  | | 0 | 0 | |  |  | | 0 | 0 | |  |
| Other |  |  | | 1 | 2.3 | |  |  | | 0 | 0 | |  |  | | 1 | 3.3 | |  |
| Witnessed | 51 | 94.4 | | 46 | 90.2 | | 13 | 81.3 | | 12 | 92.3 | | 38 | 100 | | 34 | 89.5 | | 1.000 |
| By a bystander |  |  | | 42 | 82.4 | |  |  | | 11 | 84.6 | |  |  | | 31 | 81.6 | |  |
| By the EMS |  |  | | 4 | 7.8 | |  |  | | 1 | 7.7 | |  |  | | 3 | 7.9 | |  |
| First cardiac rhythm | 53 | 98.1 | |  |  | | 16 | 100 | |  |  | | 37 | 97.4 | |  |  | | 0.627 |
| Vfib |  |  | | 33 | 62.3 | |  |  | | 12 | 75 | |  |  | | 21 | 56.8 | |  |
| pVT |  |  | | 2 | 3.8 | |  |  | | 0 | 0 | |  |  | | 2 | 5.4 | |  |
| Asystole |  |  | | 3 | 5.7 | |  |  | | 1 | 6.3 | |  |  | | 2 | 5.4 | |  |
| PEA |  |  | | 4 | 7.5 | |  |  | | 0 | 0 | |  |  | | 4 | 10.8 | |  |
| AED Shockable |  |  | | 11 | 20.8 | |  |  | | 3 | 18.8 | |  |  | | 8 | 21.6 | |  |
| AED Nonshockable |  |  | | 0 | 0 | |  |  | | 0 | 0 | |  |  | | 0 | 0 | |  |
| Prehospital resuscitation |  | |  |  | |  |  | |  |  | |  |  | |  |  | |  |  |
| Bystander CPR | 43 | | 79.6 | 33 | | 74.4 | 12 | | 75 | 9 | | 75 | 31 | | 81.6 | 24 | | 77.4 | 1.000 |
| Bystander defibrillation | 42 | | 77.8 | 12 | | 28.6 | 12 | | 75 | 3 | | 25 | 31 | | 81.6 | 9 | | 29 | 1.000 |
| Defibrillation | 53 | | 98.1 | 48 | | 90.6 | 16 | | 100 | 15 | | 93.8 | 37 | | 97.4 | 33 | | 89.2 | 1.000 |
| Drug used | 51 | | 94.4 | 18 | | 35.3 | 15 | | 93.8 | 6 | | 40 | 36 | | 94.7 | 13 | | 36.1 | 0.960 |
| Adrenaline only |  | |  | 12 | | 23.5 |  | |  | 4 | | 26.7 |  | |  | 8 | | 22.2 |  |
| Amiodarone |  | |  | 3 | | 5.88 |  | |  | 1 | | 6.7 |  | |  | 2 | | 5.6 |  |
| Adrenaline and amiodarone |  | |  | 3 | | 5.88 |  | |  | 1 | | 6.7 |  | |  | 2 | | 5.6 |  |
| Magnesium |  | |  | 1 | | 1.85 |  | |  | 0 | | 0 |  | |  | 1 | | 2.8 |  |
| Vasopressin |  | |  | 0 | | 0 |  | |  | 0 | | 0 |  | |  | 0 | | 0 |  |
| TTM | 22 | | 40.7 |  | |  | 9 | | 56.3 |  | |  | 13 | | 34.2 |  | |  | 0.830 |
| Intra-arrest |  | |  | 0 | | 0 |  | |  | 0 | | 0 |  | |  | 0 | | 0 |  |
| Prehospital after ROSC |  | |  | 5 | | 22.7 |  | |  | 1 | | 11.1 |  | |  | 4 | | 30.8 |  |
| In-hospital after ROSC |  | |  | 6 | | 27.2 |  | |  | 3 | | 33.3 |  | |  | 3 | | 23.1 |  |
| Indicated but not performed |  | |  | 4 | | 18.1 |  | |  | 2 | | 22.2 |  | |  | 2 | | 15.4 |  |
| Not indicated |  | |  | 7 | | 31.8 |  | |  | 3 | | 33.3 |  | |  | 4 | | 30.8 |  |
| In-hospital post resuscitation care |  | |  |  | |  |  | |  |  | |  |  | |  |  | |  |  |
| Coronary reperfusion attempted | 54 | | 100 | 52 | | 96.3 | 16 | | 100 | 16 | | 100 | 38 | | 100 | 36 | | 94.7 | 0.174 |
| Angiography only |  | |  | 23 | | 42.6 |  | |  | 10 | | 62.5 |  | |  | 13 | | 34.2 |  |
| PCI |  | |  | 29 | | 53.7 |  | |  | 6 | | 37.5 |  | |  | 23 | | 60.5 |  |
| Intravenous thrombolysis |  | |  | 0 | | 0 |  | |  | 0 | | 0 |  | |  | 0 | | 0 |  |

This table shows the categorical variables of patient characteristics for the overall study population, and patients with and without ICD implantation. The first column of each patient group presents the number of known data values for each variable, along with the percentage out of the total number of patients in the group. The frequency of the specified characteristics, along with the percentage out of the number of known data values in the subgroup, is provided. The comparison p-value is obtained using a Fisher’s exact test. An asterisk (*) indicates a statistical significance when p <0.05.
