# Supplementary material for: Right atrium area is associated with survival after out-of-hospital cardiac arrest: a single-center cohort study
Source: Echo Res Pract. 2025 Apr 14;12:9. doi: 10.1186/s44156-025-00072-5 (PMC11995584; doi:10.1186/s44156-025-00072-5)
Supplement: Supplementary file 3 — Supplementary Material 3: Additional file 3: Echocardiographic characteristics by survival-to-discharge. [file 44156_2025_72_MOESM3_ESM.docx]

Additional file 3: Echocardiographic characteristics by survival-to-discharge

|  | **Total (N=54)** | | | | **Survivor (N=42)** | | | | **Non-survivor (N=12)** | | | |  | | | |  |
| --- | --- | --- | --- | --- | --- | --- | --- | --- | --- | --- | --- | --- | --- | --- | --- | --- | --- |
| **Echocardiographic parameter** | **Number of known data** | | **Value from known data** | | **Number of known data** | | **Value from known data** | | **Number of known data** | | **Value from known data** | | **Comparison**  **p-value** | |  |  |  |
|  |  | **%** | **Mean**  **Median** | **1SD**  **IQR** |  | **%** | **Mean**  **Median** | **1SD**  **IQR** |  | **%** | **Mean**  **Median** | **1SD**  **IQR** |  |  |  |  |  |
| Timing of first scan | 54 | 100 | 6.56 | ±7.66 | 42 | 100 | 7.47 | ±8.07 | 12 | 100 | 3.33 | ±5.07 | 0.021*^a^ |  |  |  |  |
| (days) |  |  | 4 | 2-7 |  |  | 5.5 | 2-10 |  |  | 2 | 1-3 |  |  |  |  |  |
| Basic parameters |  |  |  |  |  |  |  |  |  |  |  |  |  |  |  |  |  |
| Body weight (kg) | 52 | 96.3 | 82.2 | ±13.0 | 41 | 97.6 | 82.0 | ±11.1 | 11 | 91.7 | 82.7 | ±19.2 | 0.887 |  |  |  |  |
| Height (cm) | 51 | 94.4 | 173 | ±8.47 | 41 | 97.6 | 173 | ±9.00 | 10 | 83.3 | 172 | ±6.05 | 0.676 |  |  |  |  |
| BSA (m^2^) | 51 | 94.4 | 1.98 | ±0.191 | 41 | 97.6 | 1.98 | ±0.167 | 10 | 83.3 | 1.98 | ±0.281 | 0.959 |  |  |  |  |
| MAP (mmHg) | 50 | 92.6 | 81.8 | ±16.2 | 40 | 95.2 | 81.4 | ±17.9 | 10 | 83.3 | 83.2 | ±6.94 | 0.283^a^ |  |  |  |  |
|  |  |  | 79 | 71.3-89.3 |  |  | 77.8 | 69.3-90.2 |  |  | 82.5 | 79-88.7 |  |  |  |  |  |
| HR (bpm) | 54 | 100 | 69.9 | ±16.8 | 42 | 100 | 68.2 | ±17.2 | 12 | 100 | 75.7 | ±14.5 | 0.083^a^ |  |  |  |  |
|  |  |  | 68 | 60-80 |  |  | 65 | 60-75 |  |  | 80.5 | 62.5-85 |  |  |  |  |  |
| LA dimensions |  |  |  |  |  |  |  |  |  |  |  |  |  |  |  |  |  |
| LA diameter (cm) | 50 | 92.6 | 3.66 | ±0.534 | 40 | 95.2 | 3.60 | ±0.483 | 10 | 83.3 | 3.89 | ±0.684 | 0.130 |  |  |  |  |
| LAV (mL) | 49 | 90.7 | 61.6 | ±21.0 | 39 | 92.9 | 59.5 | ±20.1 | 10 | 83.3 | 70.0 | 23.3 | 0.116^a^ |  |  |  |  |
|  |  |  | 28.2 | 22. |  |  | 57 | 46.5-68.6 |  |  | 66.3 | 56.7-90.1 |  |  |  |  |  |
| LAVI (mL/m^2^) | 46 | 85.2 | 30.8 | ±11.2 | 38 | 90.5 | 29.8 | ±10.9 | 5 | 41.7 | 36 | ±11.8 | 0.102^a^ | | |  |  |
|  |  |  | 28.2 | 22.7-34.6 |  |  | 27.7 | 22.4-32.8 |  |  | 38.1 | 29.0-44.4 |  | | | | |
| LV diastolic function |  |  |  |  |  |  |  |  |  |  |  |  |  | | | | |
| Mitral E (m/s) | 52 | 96.3 | 0.66 | ±0.230 | 41 | 97.6 | 0.640 | ±0.234 | 11 | 91.7 | 0.72 | ±0.213 | 0.313 | | | | |
| Mitral A (m/s) | 49 | 90.7 | 0.62 | ±0.221 | 40 | 95.2 | 0.608 | ±0.228 | 9 | 75.0 | 0.65 | ±0.199 | 0.472^a^ | | | | |
|  |  |  | 0.57 | 0.47-0.7 |  |  | 0.551 | 0.46-0.7 |  |  | 0.62 | 0.51-0.71 |  | | | | |
| Mitral E/A | 48 | 88.9 | 1.18 | ±0.654 | 40 | 95.2 | 1.19 | ±0.679 | 8 | 66.7 | 1.12 | ±0.545 | 0.968^a^ | | | | |
|  |  |  | 0.977 | 0.709-1.54 |  |  | 0.977 | 0.704-1.54 |  |  | 0.977 | 0.743-1.38 |  | | | | |
| Mitral E DT (ms) | 50 | 92.6 | 196 | ±56.0 | 39 | 92.9 | 200 | ±57.2 | 11 | 91.7 | 184 | ±52.2 | 0.422 | | | | |
| Mitral septal e’ (m/s) | 37 | 68.5 | 0.0680 | ±0.0191 | 31 | 73.8 | 0.0665 | ±0.0190 | 6 | 50.0 | 0.0753 | ±0.0199 | 0.309 | | | | |
| Mitral septal s’ (m/s) | 28 | 51.9 | 0.0802 | ±0.0261 | 24 | 57.1 | 0.0804 | ±0.0197 | 4 | 33.3 | 0.079 | ±0.0563 | 0.920 | | | | |
| Mitral septal E/e’ | 36 | 66.7 | 10.2 | ±4.86 | 31 | 73.8 | 10.2 | ±4.85 | 5 | 41.7 | 10.2 | ±5.53 | 0.894^a^ | | | | |
|  |  |  | 8.37 | 7.08-12.5 |  |  | 8.33 | 7.33-12.4 |  |  | 9.5 | 5.77-14.3 |  | | | | |
| LV Dimensions |  |  |  |  |  |  |  |  |  |  |  |  |  | | | | |
| LVESD (cm) | 53 | 98.1 | 3.64 | ±0.919 | 42 | 100 | 3.58 | ±0.825 | 11 | 91.7 | 3.86 | ±1.24 | 0.395^a^ | | | | |
|  |  |  | 3.67 | 2.98-4.06 |  |  | 3.30 | 2.90-4.04 |  |  | 4 | 2.7-4.85 |  | | | | |
| LVEDD (cm) | 53 | 98.1 | 4.98 | ±0.760 | 42 | 100 | 4.97 | ±0.590 | 11 | 91.7 | 5.04 | ±1.25 | 0.294^a^ | | | | |
|  |  |  | 5 | 4.49-5.51 |  |  | 4.95 | 4.49-5.4 |  |  | 5.52 | 4.2-6.09 |  | | | | |
| LVFS (%) | 53 | 98.1 | 27.7 | ±9.97 | 42 | 100 | 28.6 | ±9.58 | 11 | 91.7 | 24 | ±11 | 0.177 | | | | |
| LV mass index (g/m^2^) | 49 | 90.7 | 95.2 | ±28.0 | 40 | 95.2 | 93.9 | ±20.0 | 9 | 75.0 | 101 | ±52 | 0.970^a^ | | | | |
|  |  |  | 93.5 | 80.4-102.7 |  |  | 93.6 | 81.7-101.8 |  |  | 88.4 | 66.7-135.6 |  | | | | |
| LV Systolic function |  |  |  |  |  |  |  |  |  |  |  |  |  | | |  |  |
| LVOT Vmax (m/s) | 52 | 96.3 | 0.934 | ±0.191 | 41 | 97.6 | 0.943 | ±0.185 | 11 | 91.7 | 0.896 | ±0.216 | 0.435 | | |  |  |
| LVOT VTI (cm) | 52 | 96.3 | 17.9 | ±5.08 | 41 | 97.6 | 18.0 | ±5.15 | 11 | 91.7 | 17.5 | ±5.02 | 0.764 | | |  |  |
| WMS | 54 | 100 | 24.9 | ±6.19 | 42 | 100 | 24.7 | ±6.41 | 12 | 100 | 25.8 | ±5.55 | 0.505^a^ | | |  |  |
|  |  |  | 26 | 20-30 |  |  | 25.5 | 19-30 |  |  | 27 | 20-29.5 |  | | |  |  |
| WMSI | 54 | 100 | 1.57 | ±0.391 | 42 | 100 | 1.55 | ±0.406 | 12 | 100 | 1.63 | ±0.347 | 0.498^a^ | | |  |  |
|  |  |  | 1.6 | 1.3-1.9 |  |  | 1.6 | 1.2-1.9 |  |  | 1.7 | 1.3-1.8 |  | | |  |  |
| LVEF (%) | 54 | 100 | 43.9 | ±11.4 | 42 | 100 | 44.4 | ±12.0 | 12 | 100 | 42.4 | ±9.31 | 0.609 | | |  |  |
| LVGLS, peak averaged (%) | 31 | 57.4 | -10.8 | ±4.22 | 28 | 66.7 | -10.9 | ±4.14 | 3 | 25.0 | -9.39 | ±5.68 | 0.677^a^ | | |  |  |
|  |  |  | -11.4 | -14.0- -7.61 |  |  | -11.0 | -14.1- -8.0 |  |  | -12.6 | -12.8- -2.83 |  | | |  |  |
|  |  |  | **SD_p_:** | ±4.26 |  |  |  |  |  |  |  |  |  | | |  |  |
| Right heart |  |  |  |  |  |  |  |  |  |  |  |  |  | | |  |  |
| RAA (cm^2^) | 43 | 79.6 | 16.3 | ±4.99 | 35 | 83.3 | 15.2 | ±4.46 | 8 | 66.7 | 20.8 | ±4.84 | 0.003* | | |  |  |
| RAP (mmHg) | 33 | 61.1 | Count | % | 25 | 59.5 | Count | % | 8 | 66.7 | Count | % | 0.250 | | |  |  |
| 0-5 |  |  | 23 | 69.7 |  |  | 19 | 76.0 |  |  | 4 | 50.0 |  | | |  |  |
| 5-10 |  |  | 4 | 12.1 |  |  | 3 | 12.0 |  |  | 1 | 12.5 |  | | |  |  |
| 15 |  |  | 6 | 18.1 |  |  | 3 | 12.0 |  |  | 3 | 37.5 |  | | |  |  |
| RVSP (mmHg) | 20 | 37.0 | 36.1 | ±13.2 | 15 | 35.7 | 36.6 | ±14.3 | 5 | 41.7 | 34.7 | ±10.3 | 0.784 | | |  |  |
| TAPSE (cm) | 34 | 63.0 | 1.95 | ±0.500 | 28 | 66.7 | 2.00 | ±0.46 | 6 | 50.0 | 1.71 | ±0.638 | 0.200 | | |  |  |
| FAC (%) | 33 | 61.1 | 41.7 | ±10.2 | 28 | 66.7 | 41.6 | ±10.2 | 5 | 41.7 | 41.9 | ±12.0 | 0.956 | | |  |  |
| Hemodynamics |  |  |  |  |  |  |  |  |  |  |  |  |  | | |  |  |
| SV (mL) | 49 | 90.7 | 60.0 | ±19.7 | 39 | 92.9 | 60.8 | ±19.7 | 10 | 83.3 | 56.9 | ±20.3 | 0.576 | | |  |  |
| SVI (mL/m^2^) | 46 | 85.2 | 31.0 | ±10.7 | 38 | 90.5 | 31.1 | ±10.2 | 8 | 66.7 | 30.6 | ±13.9 | 0.900 | | |  |  |
| CO (mL/min) | 49 | 90.7 | 4070 | ±1280 | 39 | 92.9 | 4000 | ±1130 | 10 | 83.3 | 4330 | ±1810 | 0.468 | | |  |  |
| CI (mL/min/m^2^) | 46 | 85.2 | 2090 | ±766 | 38 | 90.5 | 2050 | ±625 | 8 | 66.7 | 2280 | ±1290 | 0.450 | | |  |  |

This table shows the echocardiographic characteristics for the overall study population, survivors and non-survivors. The first column of each patient group presents the number of known data values for each variable, along with the percentage out of the total number of patients in the group. The mean and the SD for each variable in each group are provided. When the SW test p-value is <0.05 in any subgroup, the median and the IQR are also given. The pooled standard deviation (SD_p)_ is also provided for LVGLS. The comparison p-value for RAP is obtained using a Fisher’s exact test. Other comparison p-values are obtained using the t-test for independent samples, or for values marked with (^a^), using the exact probability from a Mann Whitney U test. An asterisk (*) indicates a statistical significance when p <0.05. New abbreviations used in this table include: BSA (body surface area), CO (cardiac output), CI (cardiac index), DT (deceleration time), E (peak early inflow velocity), E/A (E to late inflow velocity ratio), e’ (peak early annular velocity), E/e’ (E to e’ ratio), FAC (fractional area change), HR (heart rate), LA (left atrium), LAV (LA volume), LAVI (LAV index), LVEDD (LV end-diastolic diameter), LVESD (LV end-systolic diameter), MAP (mean arterial pressure), RAA (right atrium area), RAP (right atrium pressure), RVSP (RV systolic pressure), s’ (peak systolic annular velocity), SV (stroke volume), SVI (SV index), TAPSE (tricuspid annular plane systolic excursion), Vmax (peak velocity).
